# Associations between Traumatic Brain Injury, Drug Abuse, Alcohol Use, Adverse Childhood Events, and Aggression Levels in Individuals with Foster Care History

**DOI:** 10.1089/neur.2020.0032

**Published:** 2020-12-10

**Authors:** Michael D. Cusimano, Stanley Zhang, Grace Huang, David Wolfe, Melissa Carpino

**Affiliations:** ^1^ Injury Prevention Research Office, Division of Neurosurgery, St. Michael's Hospital, Toronto, Ontario, Canada.; ^2^ Department of Surgery, University of Toronto, Toronto, Ontario, Canada.; ^3^ Dalla Lana School of Public Health, University of Toronto, Toronto, Ontario, Canada.; ^4^ Center for School Mental Health, Faculty of Education, Western University, London, Ontario, Canada.

**Keywords:** adverse childhood events, aggression, foster children, neuroimaging, substance use, traumatic brain injury

## Abstract

Nearly 50,000 Canadian children live in foster care. Compared with their peers, foster children experience greater independence and decreased guidance, predisposing them to harmful exposures such as traumatic brain injury (TBI), illicit drugs, and alcohol. Foster children also report a higher level of childhood abuse compared with the general population. This study aimed to: 1) investigate substance/alcohol use disorder, adverse childhood events (ACE), TBI, aggression levels, and the difference between normalized percentages of brain regions of interest (ROIs) in a sample of Canadian youths with and without foster care history; 2) determine the prevalence of substance/alcohol use disorder, ACE, and aggression levels within individuals with foster care history when stratified by likelihood of TBI; and 3) determine the significant correlates of elevated aggression levels within this population. Participants completed standardized questionnaires that measured the prevalence of TBI, substance and alcohol use disorder, ACE, and aggression. Magnetic resonance imaging (MRI) was used to measure differences in brain ROI. Regression and network analysis were used to study interactions between variables. Seventy-four participants (51 individuals with foster care history and 23 age-matched controls from the general population) completed standardized questionnaires. Fifty-five of these individuals (39 foster participants and 16 controls) underwent brain MRI. Foster participants had higher prevalence of substance use disorder (*p* < 0.001), alcohol use disorder (*p* = 0.003), ACE (*p* < 0.001), and elevated aggression levels (*p* < 0.001) than healthy controls. No significant difference was found among brain ROI. The prevalence of TBI in foster participants was 65%. Foster participants with moderate or high likelihood of TBI exposure had higher levels of drug use and aggression than those with no or low likelihood of exposure. Brain volumes were not associated with substance/alcohol use disorder or ACE. No significant associations were found between aggression levels and the studied variables.

## Introduction

Currently, there are over 47,000 Canadian,^1^ 690,000 American,^2^ and 56,160 British^3^ children living in foster care under the legal responsibility of the government or other family members rather than their biological parents. Compared with their peers, the greater independence and decreased guidance experienced by foster children predisposes them to harmful exposures such as traumatic brain injury (TBI), illicit drugs, and alcohol.^4–7^ Foster care children also report a higher level of childhood abuse compared with the general population.^8^ However, there are some inconsistencies with the aforementioned results attributed to modest sample sizes and varying methods of measuring outcomes of interest.

TBI has been linked to elevated risks of substance use disorder,^9^ alcohol use disorder,^10^ and aggression,^9,11,12^ whereas increased aggression is associated with substance use^13^ and childhood physical abuse and internalizing behavior.^14^ Further, elevated levels of aggression are noted to be associated with reduced amygdala, hippocampal,^15^ and prefrontal cortical volume in alcoholics,^16^ psychiatric patients, and healthy populations.^11,13–15^ Aggression is also linked to increased white matter volumes in occipital and parietal lobes and increased gray matter volume in right cerebellum.^17^ However, results are inconsistent as some studies have found increased aggression associated with increased volumes,^18^ whereas other studies reported null findings.^17^ To date, these relationships have not been explored among those with a history of foster care; but one may consider that those with a history of foster care might have higher levels of adverse outcomes such as aggression and substance/alcohol use disorders and associated reduced volumes of amygdala, hippocampal, and prefrontal cortical volumes.

To our knowledge, no studies have simultaneously examined TBI, substance abuse, alcohol use, childhood abuse, and aggression in a foster population using neurological imaging. Thus, our study aimed to: 1) investigate substance use disorder, alcohol use disorder, adverse childhood events (ACE), TBI, aggression levels, and the difference between normalized percentages of brain regions of interest (ROIs) in a sample of Canadian youths and young adults with and without foster care history; 2) determine the prevalence of substance use disorder, alcohol use disorder, ACE, and aggression levels within individuals with foster care history when stratified by likelihood of TBI exposure; and 3) determine the significant correlates of elevated aggression levels within this population. We hypothesized that individuals with foster care history will have a higher prevalence of TBI, substance/alcohol use disorders, and childhood abuse; higher aggression levels; lower volumes of amygdala, hippocampus, and cortex; and higher volumes of white and gray matter than controls.

## Methods

### Study design and ethics approval

A cross-sectional investigation was used to collect data on current and former foster children living in Toronto, Ontario, Canada. The research was approved by the Research Ethics Board at St. Michael's Hospital, Toronto, Ontario, Canada.

### Participants

The inclusion criteria for foster care participants consisted of being an English speaker, being at least 16 years old, and having been registered at a child welfare location. Foster participants were identified and recruited through child protective services (the Catholic Children's Aid Society), community youth organizations (Yonge Street Mission's Evergreen Center for Street Youth and Covenant House Toronto), and referral from other participants. To encourage recruitment, we printed advertisements and posted them on bulletin boards at the various youth organizations, schools, and shelters.

A control group was recruited from the general population from St. Michael's Hospital, University of Toronto, local high schools, and referrals from other participants. Controls were matched to cases (participants with foster care history) based on age. In turn, controls were required to be English speakers, at least 16 years old, have no foster care involvement, and have never had a self-reported TBI. All participants were compensated in an amount approved by the Research Ethics Board (which was equal to the provincial minimum wage per hour plus travel by public transit), and according to the degree of time commitment. In general, participants who completed all tests and underwent magnetic resonance imaging (MRI) received more compensation than those who did not undergo MRI because of the longer time required for the MRI component, which was conducted in addition to the other components of the study.

### Procedure

Prior to being enrolled in the study, all participants provided written informed consent. Once enrolled, participants were asked to complete a series of questionnaires under the supervision of a research assistant. Those who were eligible and consented for MRI were scanned according to the protocol described below.

### Measures

#### Demographics and TBI exposure

All participants (51 foster participants and 23 controls) completed the General Information Questionnaire, which gathered information on age, sex, gender, education level, income, history of psychiatric disorders, ethnicity, and self-reported history of TBI. TBI was defined as a “Yes” response to the question: “Have you ever had an injury to the head which knocked you out or at least left you dazed, confused, or disoriented?”

#### Likelihood of TBI exposure

The Brain Injury Screening Questionnaire (BISQ)^19^ was used to determine the likelihood of lifetime TBI exposure.^20^ This questionnaire had three sections: TBI history, symptoms, and other health conditions. Each participant received a final BISQ score ranging from 0 (no exposure), 1 (low), 2 (moderate), to 3 (high probability) of TBI exposure. Because the BISQ score is also associated with severity of lifetime TBI, a higher score indicated a more severe case.^19^

#### Substance use

The Drug Abuse Screening Test (DAST) is a validated method for identifying individuals who abuse psychoactive drugs and classifying the severity of misuse.^21^ The data from this test were self-reported with the participant marking either a “Yes” or “No” response to each question. This screening test addressed multiple substance use and problems caused by drug abuse including employment, medical conditions, and family and social relationships. Each response indicating a tendency toward substance use added 1 point to the total score. A score of 6 or higher was considered a positive screen for substance use disorder.

#### Alcohol use

The Alcohol Use Disorders Identification Test (AUDIT) is a validated 10-item questionnaire that asks about the frequency or extent of alcohol-related activities,^22^ and is scored similarly to the DAST for a maximum score of 40. This test covers topics including frequency of drinking and effects on employment, mental health, and friends and family. A score above 8 suggested a positive screen for alcohol use disorder, whereas a score of 20 or more indicated extremely problematic drinking.

#### Adverse childhood events

The Adverse Childhood Experiences (ACE) Questionnaire is a 10-item questionnaire that asks about exposure to domestic violence, sexual abuse, physical abuse, verbal abuse, and drug abuse and mental health in caretakers. Each exposure added 1 point to the overall ACE Questionnaire score for a maximum score of 10.

#### Aggression

The Buss Perry Aggression Questionnaire (BPAQ) contains 29 questions that aim to measure physical aggression, verbal aggression, anger, and hostility.^23^ For each question, participants were asked to rank their likelihood of performing each behavior on a scale from 1 to 7. The points from each question were tallied for a maximum score of 63 in physical aggression, 35 in verbal aggression, 49 in anger, 56 in hostility, and a total score of 203. A higher score indicated more aggressive behavior.

#### MRI images

Brain images were obtained using the 3.0 Tesla research-dedicated MRI system (MR750, GE Healthcare, Waukesha, WI, USA) at Sunnybrook Health Center in Toronto. A standard 12-channel head phased-array coil was used. MRI protocol consisted of T1-weighted fast gradient echo images (in-plane resolution = 0.9 mm × 0.9 mm; field of view [FOV] = 220 mm × 165 mm; slice thickness = 1.4 mm; flip angle = 15 degrees; repetition time [TR] = 8.2 msec; echo time [TE] = 3.2 msec), axial proton-density weighted T2 (PD-T2) images (resolution = 0.9 mm × 0.9 mm × 3.0 mm; FOV = 220 mm × 165 mm; slice thickness = 3 mm; flip angle = 90 degrees; TR = 2900 msec; TE = 22.2/88.8 msec), axial FLAIR (fluid attenuated inversion recovery) images (resolution = 0.9 mm × 1.1 mm × 3.0 mm; FOV = 220 mm × 200 mm; slice thickness = 3 mm; flip angle = 90 degrees; TR = 9950 msec; TE = 96 msec), and axial gradient recalled echo (GRE) images (resolution = 0.8 mm × 1.0 mm × 3.0 mm; FOV = 200 mm × 200 mm; slice thickness = 3mm; flip angle = 20 degrees; TR = 784 msec; TE = 35 msec).

#### MRI data processing

To calculate regional brain volumes, the T1-weighted images of both the foster and control groups were analyzed using the fully automated FreeSurfer Pipeline version 5.3.0.^24^ Specifically, the volume-based stream was used, which consists of six stages: affine registration with the Montreal Neurological Institute Atlas (MNI305), initial volumetric labeling, B1 bias field correction, skull stripping, high-dimensional non-linear volumetric alignment to the MNI305, and eventually data labeling (segmentation). The program divides each T1-weighted image into 65 substructures and calculates the volume of each. Of the 65 brain regions computed by FreeSurfer, structures related to aggression were specifically examined, including the amygdala, hippocampus, cortex, cortical white matter, and total gray matter.^25–27^ Due to the limited areas generated by FreeSurfer, total cortex volume was examined instead of specific lobular volume. The volumetric data were normalized using the well-accepted proportion method by dividing the ROI volume by total intracranial volume (ICV).^28^

### Statistical analysis

We used a chi-squared or Fisher's exact test to compare the demographic variables between the foster and control group. The prevalence of TBI, substance use disorder, alcohol use disorder, and ACE Questionnaire score of the foster and control group were calculated by dividing the number of participants with the outcome of interest by the total number of participants in the group. We used a chi-squared test to compare the rate of substance use disorder and alcohol use disorder between the foster and control groups. Questionnaire scores between the foster and control group, volumetric differences in the normalized percentage and volume of brain structures of interest between the foster and control group, and questionnaire scores in foster groups stratified by likelihood of TBI exposure were all compared using a two-tailed *t* test.

To investigate the interplay of the studied factors, a network analysis was conducted using Cytoscape to visualize the undirected, binary interactions.^29^ Nodes represented variables including questionnaire scores, foster care history, and volumetric data. Edges represented the interaction between variables, with a thicker edge representing a stronger association. We selected only the interactions with a Pearson's coefficient of higher than 0.4.

An ordinary least square (OLS) regression was used to investigate significant correlates of elevated aggression levels within the sample. The independent variable was likelihood of TBI history and the dependent variable was BPAQ score. Confounding variables included in the model were age, gender, drug abuse (DAST score), alcohol use (AUDIT score), ACEs (ACE Questionnaire score), and normalized amygdala, hippocampus, and cortex volumes. Only individuals with complete questionnaire and volumetric data (39 foster participants and 16 controls) were included in the regression.

## Results

### Demographic information

As shown in Table 1, 74 participants (51 foster participants and 23 controls) were between 16 and 30 years of age with a mean age of 21.6 years (standard deviation [SD] = 3.18) in the foster group and 21.1 (SD = 3.28) in the control group. A chi-square test showed no significant difference in age and sex between the two groups; however, a larger proportion of controls were enrolled in post-secondary education and had higher incomes. White or Caucasian (47%) was the most prevalent ethnicity in the foster group and Asian or Asian Canadian (47%) was the most prevalent ethnicity in the control group. On average, foster participants stayed in foster care for 5.9 years (SD = 4.2).

**Table 1.** Demographic Characteristics of Individuals with Foster Care History and Controls (*n* = 74)

		Foster participants (*n* = 51)*N *(%)	Control participants (*n* = 23)*N *(%)	P
Age (years)				0.927
	16–18	11 (0.22)	6 (0.26)	
	19–22	19 (0.37)	7 (0.30)	
	23–26	17 (0.33)	9 (0.39)	
	27–30	4 (0.08)	1 (0.04)	
				
Sex				0.080
	Male	30 (0.59)	8 (0.35)	
	Female	21(0.41)	15 (0.65)	
				
Education				<0.001
	Junior high school or less	13 (0.25)	3 (0.13)	
	Some high school	1 (0.02)	0 (0.00)	
	High school/GED	30 (0.59)	5 (0.22)	
	Some college	4 (0.08)	1 (0.03)	
	College	1 (0.02)	1 (0.04)	
	Some university	0 (0.00)	6 (0.26)	
	Bachelor's degree	1 (0.02)	5 (0.22)	
	Some graduate classes	1 (0.02)	2 (0.09)	
				
Gross income				<0.0001
	Less than $10,000	22 (0.43)	2 (0.09)	
	$10 000–$20,000	13 (0.25)	2 (0.09)	
	$20 000–$40,000	6 (0.12)	6 (0.26)	
	$40 000–$60,000	0 (0.00)	3 (0.13)	
	$60 000–$100,000	0 (0.00)	3 (0.13)	
	More than $100,000	0 (0.00)	3 (0.13)	
	Declined to answer	10 (0.20)	4 (0.17)	
				
TBI history				
	TBI	33 (0.65)	0 (0.00)	
	No TBI	18 (0.35)	23 (1.00)	
				
Ethnicity				<0.0001
	White, Caucasian	24 (0.47)	4 (0.17)	
	Asian, Asian-Canadian	3 (0.06)	11 (0.48)	
	Black, African, African-American	5 (0.1)	1 (0.04)	
	Hispanic, Latino, Latina	1 (0.02)	3 (0.13)	
	Caucasian	5 (0.10)	1 (0.04)	
	Native American	6 (0.12)	0 (0.00)	
	Other	2 (0.04)	1 (0.04)	
	Declined to answer	5 (0.10)	0 (0.00)	
	Missing data	0 (0.00)	2 (0.09)	
				
Psychiatric disorder				0.090
	Yes	10 (0.20)	1 (0.04)	
	No	35 (0.69)	20 (0.87)	
	Missing data	6 (0.12)	2 (0.09)	
				
Length of foster care				
	Less than 1 year	3 (0.06)	Not applicable	
	1 to 5 years	13 (0.25)		
	5 to 10 years	13 (0.25)		
	10 or more years	4 (0.08)		
	Declined to answer	18 (0.35)		

TBI, traumatic brain injury.

### Comparison of substance use, alcohol use, adverse childhood events, and aggression levels between foster and control groups

**FIG. 1.**
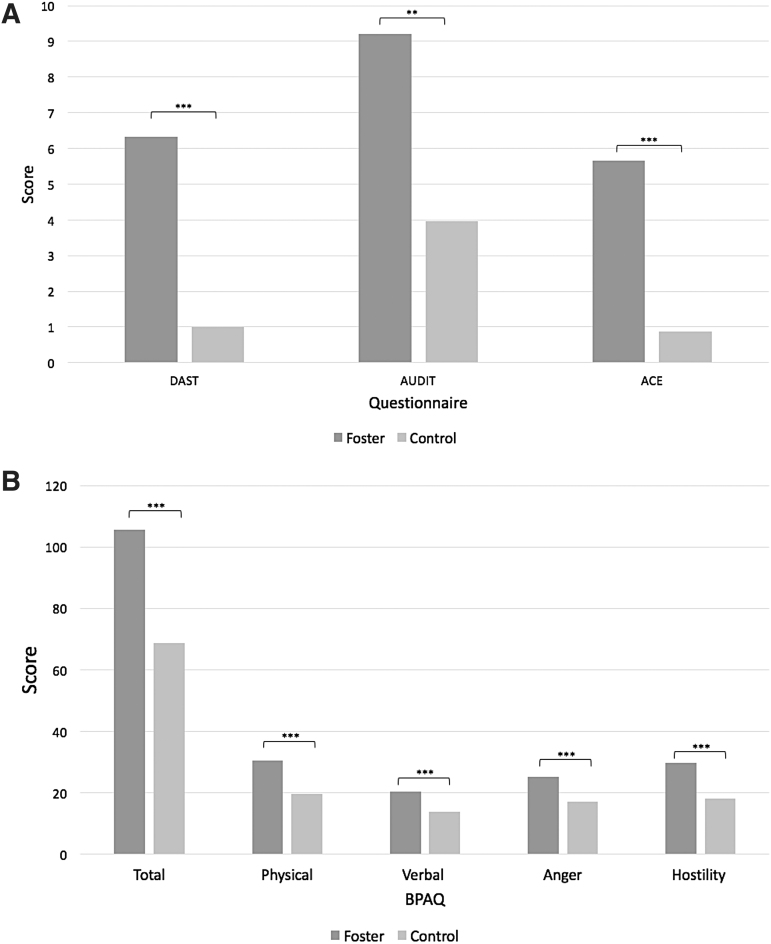
**(A)** DAST, AUDIT, and ACE scores in foster and control groups. **(B)** BPAQ total and subsection scores in foster and control groups. **indicates significance of *p* < 0.05; ***indicates significance of *p* < 0.001. ACE, Adverse Childhood Experiences Questionnaire; AUDIT, Alcohol Use Disorders Identification Test; BPAQ, Buss Perry Aggression Questionnaire; DAST, Drug Abuse Screening Test.

Forty-nine foster participants and 22 controls completed the DAST, 51 foster participants and 23 controls completed the AUDIT, 46 foster participants and 19 controls completed the ACE Questionnaire, and 51 foster participants and 22 controls completed the BPAQ. In comparison to their control group counterparts, foster group participants had significantly higher levels of substance use, alcohol use, ACEs, and aggression levels. The average DAST score was 6.33 (SD = 4.44) in the foster group and 1.00 (SD = 3.14) in the control group (*p* < 0.001). Foster participants also had a higher prevalence of substance use disorder (47% compared with 5%, *p* < 0.001) than controls. The average AUDIT score was 9.22 (SD = 8.84) in the foster group and 3.96 (SD = 5.41) in the control group (*p* = 0.003). Foster participants had a higher prevalence of alcohol use disorder than controls (49% compared with 13%, *p* = 0.002). In terms of ACEs, the average ACE Questionnaire score in the foster group was 5.66 (SD = 3.42) and it was 0.88 (SD = 1.32) in the control group (*p* < 0.001). The average total BPAQ score was 105.67 (SD = 39.37) in the foster group and 68.59 (SD = 16.83) in the control group (*p* < 0.001). Within the subsections of the BPAQ, the foster group scored significantly higher than the control group for BPAQ physical score (*p* < 0.001), verbal score (*p* < 0.001), anger score (*p* < 0.001), hostility score (*p* < 0.001), and total BPAQ score (*p* < 0.001). DAST, AUDIT, and ACE Questionnaire scores are summarized in Figure 1A and BPAQ scores are shown in Figure 1B.

#### Volumetric difference in ROI between foster and control groups

In total, 55 participants (39 foster participants and 16 controls) underwent brain MRI. No significant differences were observed in regional brain volumes of the amygdala, hippocampus, and cortex between the two groups.

### Substance use, alcohol use, aggression levels, and adverse childhood events in foster groups stratified by BISQ scores

Based on the General Information Questionnaire, the prevalence of TBI within the foster population was 65%. Similarly, the BISQ scores indicated that 62% of the foster population had a significant probability of TBI (numbers are not identical due to the reduced sample of participants who completed the questionnaire). The distribution of BISQ scores for the foster group and control group is shown in Table 2.

**Table 2.** Distribution of BISQ Scores for the Foster and Control Groups (*n* = 56)

BISQ score	Foster (*n* = 47)*N *(%)	Control (*n* = 9)*N *(%)
0	18 (0.38)	7 (0.78)
1	16 (0.34)	2 (0.22)
2	9 (0.19)	0 (0.00)
3	4 (0.09)	0 (0.00)

BISQ, Brain Injury Screening Questionnaire.

**FIG. 2.**
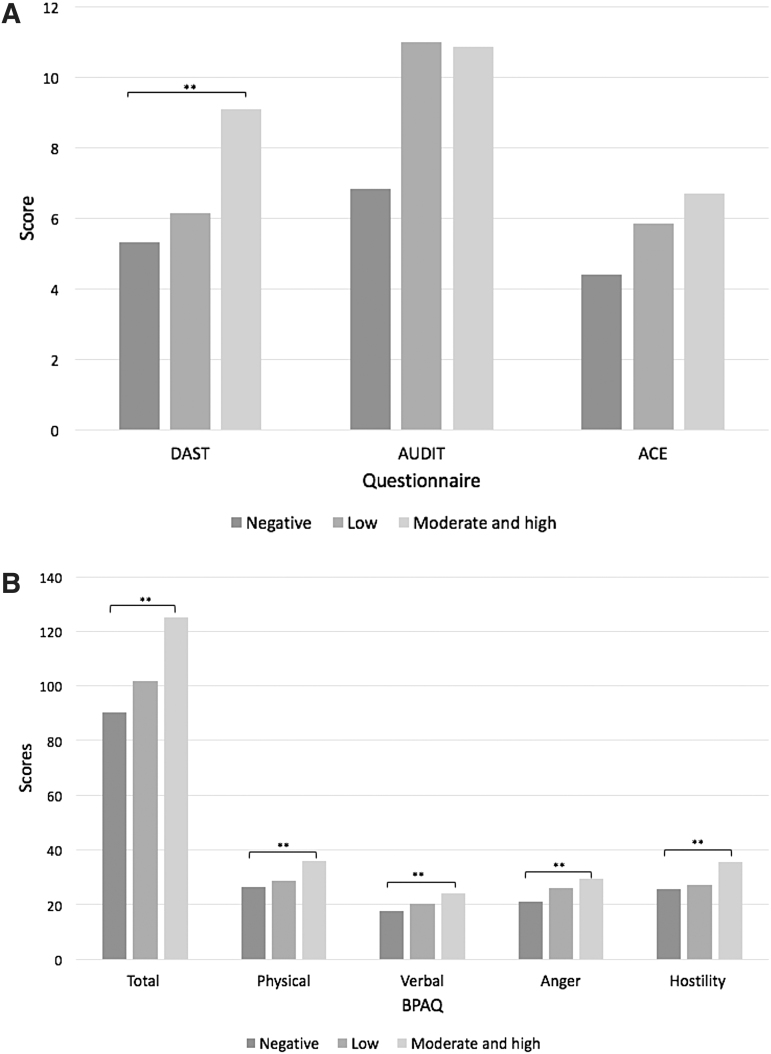
**(A)** DAST, AUDIT, and ACE score within the foster group, stratified by BISQ scores. **(B)** BPAQ total and subsection scores within foster group, stratified by BISQ scores. **indicates significance of *p* < 0.05. ACE, Adverse Childhood Experiences Questionnaire; AUDIT, Alcohol Use Disorders Identification Test; BISQ, Brain Injury Screening Questionnaire; BPAQ, Buss Perry Aggression Questionnaire; DAST, Drug Abuse Screening Test.

As illustrated in Figure 2A and 2B, within the foster group participants, when stratified by likelihood of experiencing a TBI, a higher likelihood of TBI was associated with higher DAST score, AUDIT score, ACE Questionnaire score, and BPAQ total and subsection scores. Further, it was found that foster participants with a moderate and high likelihood of TBI exposure also had significantly higher DAST scores (*p* = 0.008; Fig. 2A) as well as higher BPAQ total (*p* = 0.006), physical (*p* = 0.018), verbal (*p* = 0.044), anger (*p* = 0.029), and hostility scores (*p* = 0.032) than foster participants with a lower probability of TBI exposure (Fig. 2B). In terms of AUDIT and ACE Questionnaire scores, no significant difference was found between participants with a moderate and high likelihood of TBI exposure compared with those with no TBI exposure (*p* = 0.262 and *p* = 0.085, respectively; Fig. 2A).

### Interaction of studied variables

**FIG. 3.**
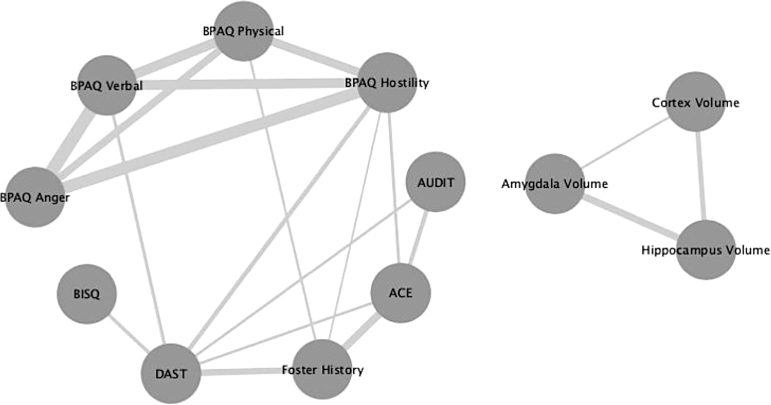
Network analysis that visualizes the relationship between the studied variables. The nodes represent questionnaire scores, foster history, and brain volume, whereas the thickness of the edges represent strength of association. All edges have a Pearson's coefficient of greater than 0.4. ACE, Adverse Childhood Experiences; AUDIT, Alcohol Use Disorders Identification Test; BISQ, Brain Injury Screening Questionnaire; BPAQ, Buss Perry Aggression Questionnaire; DAST, Drug Abuse Screening Test.

Figure 3 presents a complex web of association between the studied variables. Foster care history was correlated with higher levels of drug use, physical aggression, hostility, and childhood abuse. Drug use was correlated with a greater probability of TBI exposure, verbal aggression, hostility, alcohol use, and childhood abuse. Alcohol use was additionally correlated with childhood abuse. Strong correlations, as shown by thicker edges, were observed between the different types of aggression (physical, verbal, anger, and hostility), foster history with drug use, foster history with childhood abuse, and amygdala volume with hippocampus volume. ROI volumes were not connected with questionnaire scores, suggesting a very weak or non-existent relationship between brain volumes and drug use, alcohol use, ACEs, and aggression.

We used a linear regression model to find significant predictors of elevated aggression levels. Variables included in the model were likelihood of TBI, age, gender, alcohol use, drug abuse, and normalized brain regions of the hippocampus, amygdala, and cortex. No significant associations were found.

## Discussion

### High prevalence of TBI, drug abuse, alcohol use, adverse childhood events, and elevated aggression levels in foster participants

Our study found that 65% of the foster participants reported a history of TBI, which is much higher than the 5–38% prevalence found in the general population.^30^ Additionally, compared with their control group counterparts, foster participants had a higher prevalence of substance use disorders, alcohol use disorders, ACEs, and higher levels of aggression. Given the increased occurrence of negative health outcomes experienced by individuals within the foster care system, these findings highlight the need for additional support services for this vulnerable population.

The results of our study are consistent with other studies that reveal a higher prevalence of TBI in foster populations than in the general population.^5,7^ However, the TBI prevalence of 65% found in our study is noticeably higher than the 43% found in a study conducted by Mackelprang and colleagues.^5^ It is possible that this discrepancy is attributable to the different sample population, as Mackelprang and colleagues included former foster children as well as the homeless. As well, the higher prevalence of problematic drug and alcohol use in the foster population compared with the general population found in our study is consistent with literature.^31,32^ However, our control group had a much lower prevalence of drug and alcohol use than those illustrated in previous studies of the general population. This may be attributed to the different methods used to measure the outcome of interest. Moreover, our results support the findings of existing research that suggest that foster children experience more abuse, both physical and sexual, and maltreatment than the general population, much of which often occurs during their developmental years.^33,34^

It is often debated whether genetics (nature) or environment (nurture) plays a more prominent role in childhood development of foster children. We believe that both nature and nurture are important. Our study showed higher associations of TBI, substance/alcohol use disorders, and aggression in foster children than controls, and supports prior research showing that foster care children report a higher level of childhood abuse compared with the general population.^8^ Although some might argue that this suggests that the environment (nurture) plays a larger role than genetics in the life trajectory of foster children, our work has not explicitly explored the role of genetics. However, recently, a study found that animals raised in abusive environments have epigenetic changes that can be passed from one generation to the next.^35^ We believe that a commonality linking adverse events and risky health behaviors such as alcohol and substance use is the frequent occurrence of adverse early childhood events. In fact, other data from the present study (unpublished) derived from transcripts of life experiences of these individuals indicates that adverse experiences are often sustained at the hands of family members.

Early life exposure to adverse childhood experiences such as trauma, abuse, or maltreatment has been linked to developmental delays and mental health problems.^34^ Childhood neglect and abuse is also one of the most prominent predisposing factors to TBI^36^ as it can lead to increased risk of sustaining injuries or experiencing the persistent lingering effects of TBI. Individuals who sustained a TBI also report greater functional restrictions and disabilities compared with controls,^37^ which can possibly also contribute to stress and risk of further injury. Due to adverse experiences in their youth, foster children may also be more prone to use drugs as a coping mechanism, or may even be exposed to contexts where drugs are prevalent. Existing literature has also demonstrated that childhood trauma is related to an earlier age of substance use disorders and a greater number of years diagnosed with substance use disorders.^33^ Additionally, it has been observed that children with emotional difficulties and physical abuse also present higher levels of aggression.^14^

There is little doubt that there is a very complex web of interaction between TBI, childhood abuse, substance abuse, and aggression within the foster population. Our findings suggest that adverse early experiences play a critical role in these interactions and the development of later adverse health and social outcomes. To us, this suggests that these events adversely influence brain development at critical times, influence the resilience of individuals over time, and can subject individuals to risky environments where they are at risk for future neurological, physical, mental, and social injury.

### Neurological imaging

The neuroimaging analysis demonstrated no significant difference between the amygdala, hippocampus, and cortex volumes between the foster and control groups. In the existing literature, findings regarding volumetric data between individuals with foster care history and the general population are inconsistent. One previous study showed no differences of amygdala and hippocampal volume between previously institutionalized children and a control group,^38^ whereas another study reported smaller left hippocampal volume and larger right amygdala volume in the previously institutionalized population.^39^ Possible explanations as to why our study did not replicate previous findings may include the limited sample size, the high prevalence of psychiatric disorders (specifically depression), and other differences between study groups. For example, depression before the age of 18 has been found to be associated with decreased volume in the subgenual prefrontal cortex, and major depressive disorder has been correlated with lower gray matter volume in the cingulate gyrus.^40^ In our study, among the foster population, 20% reported having psychiatric disorders with the most prevalent disorder being depression, potentially affecting our observations.

### Web of interaction between studied variables

Our study found that there is a complex web of interaction between foster care history, adverse early childhood events, drug use, alcohol use, aggression, and TBI. However, unlike previous studies that have found an association between TBI and increased physical and verbal aggression,^41^ and a link between TBI history and increased risk of offending behavior,^12^ none of the variables in our analyses were singly responsible for increased aggression levels. Our network analysis clearly demonstrated a unique interplay of foster history and early childhood experiences, drug use, and hostility. Although TBI is important, according to our results, its role is less central to the interplay of outcomes than these other variables. Our study did not suggest an association between ROI and aggression levels, but the literature proposes that such an association may exist. Disruptions to neural circuits and injuries to the amygdala, frontal cortex, and hippocampus may cause mood changes due to biochemical changes.^42^ For example, an increase in dopamine and a decrease in serotonin levels is known to be associated with agitation and aggression.^43^ Although research in this area has yielded inconsistent results,^42^ our complex networks of interactions suggest that a change in neural function, which in our results manifested by aggression, may play the key connector function between this factor in this complex network.

### Limitations

This study has several limitations. The small sample size may increase susceptibility to outliers and reduces the statistical power. Additionally, study participants were only recruited from the Catholic Children's Aid Society, Yonge Street Mission, local high schools, and referrals, which allows for potential selection and sampling bias. Both of these issues may affect the reliability and generalizability of our findings. Although age and gender were similar between foster and control groups, differences in education level, income level, and ethnicity existed, which are factors that can influence the degree of differences seen in drug and alcohol abuse between foster children and controls.^44^ Thus, the difference observed between the two groups may, in part, be due to confounding factors.

Another potential limitation is that data regarding TBI history, drug use, alcohol use, and aggression were self-reported and likely suffer from under- and over-reporting as participants may have chosen to downplay or exaggerate their experiences. As well, the high prevalence of TBI in the foster group may have led to their desire to join the study and obtain a brain MRI. We also did not report on the age of TBI occurrence and severity of TBI, which could have influenced brain volume, because individuals who are older and have suffered a more severe TBI will have reduced gray matter and white matter volumes, respectively.^45^ Lastly, it is important to indicate that we have merely reported associations. Further information on temporality of these associations and confounders is required to determine causation.

### Implications

Overall, our results suggest that foster children experience worse outcomes in a vast array of areas. As a result, improvements made to foster care programs that help monitor physical and mental health in this population, and educational programs that better inform foster children of the risks of substance and alcohol use, may play an important role in supporting this population. Due to the complex interaction between variables, single focused initiatives will be less effective than multi-dimensional approaches. Currently, interventions generally focus on school performance, mental health, or behavioral management.^46–48^ Interventions should ideally also undergo rigorous randomized controlled studies with larger sample sizes to evaluate the effectiveness of these interventions. Interventions that influence the occurrences of critical adverse early childhood events should be strongly considered in addition to means to prevent and reduce the burden of physical injury such as TBI in childhood, as well as other forms of adverse events such as neglect and abuse. Additionally, future improvements to foster care programs should consider incorporating the role of family members due to the possibility of foster children eventually returning to their biological family.^49^

### Future directions

Future studies should analyze the relationship between the outcomes explored in this study with a larger sample size, account for more confounding factors, and include an exploration of the temporal sequences of events and the network of events we have identified. Due to the inconsistency regarding volumetric findings, more attention should be directed toward investigating the relationship between foster care history and the structure and function of different brain regions such as the amygdala, hippocampus, and regions of the cortex.

## Conclusion

Individuals with foster care history experience a greater number of adverse early childhood events, potentially making them more susceptible to deleterious outcomes including TBI, drug abuse, and alcohol use disorder, and they have higher levels of aggression than the general population. Among individuals exposed to foster care, those with a higher probability of TBI have worse outcomes. The culmination of these factors can contribute to poorer health outcomes for this vulnerable population.

## Abbreviations Used

ACE: adverse childhood events
ACE Questionnaire: Adverse Childhood Experiences Questionnaire
AUDIT: The Alcohol Use Disorders Identification Test
BISQ: Brain Injury Screening Questionnaire
BPAQ: Buss Perry Aggression Questionnaire
DAST: Drug Abuse Screening Test
FLAIR: fluid attenuated inversion recovery
FOV: field of view
GRE: gradient recalled echo
ICV: intracranial volume
MNI305: Montreal Neurological Institute Atlas
MRI: magnetic resonance imaging
OLS: ordinary least square
PD-T2: proton-density weighted T2
ROI: region of interest
SD: standard deviation
TBI: traumatic brain injury
TE: echo time
TR: repetition time
